# Interactions between mothers and infants: Impact of maternal anxiety

**DOI:** 10.1016/j.infbeh.2006.08.005

**Published:** 2007-02

**Authors:** Rosemary Nicol-Harper, Allison G. Harvey, Alan Stein

**Affiliations:** a Section of Child and Adolescent Psychiatry, University of Oxford, Warneford Hospital, Oxford OX3 7JX, United Kingdom; b Department of Psychiatry, University of Oxford, Oxford, United Kingdom; c Department of Psychology, University of California, Berkeley, United States

**Keywords:** Maternal anxiety, Postnatal, Mother–infant interactions, Sensitive responsiveness

## Abstract

The aim of the study was to examine the impact of anxiety in the postnatal year on maternal contribution to mother–infant interaction. Participants were 32 mothers with high anxiety and 32 mothers with low anxiety, when their infants were aged 10–14 months. Mother–infant interaction was videotaped during a standardized play situation and coded blind to group status. High trait anxiety mothers showed less sensitive responsivity (*p* < .05) and reduced emotional tone (*p* < .05) during interaction. When participants scoring high in depressive symptomatology were removed for a subgroup analysis, the same pattern of results was obtained, suggesting that the observed differences in mother–infant interaction were due to group differences in anxiety.

There is good evidence that parental psychological disorder increases the risk of disturbance in child development (Merikangas, Dierker, & Szatmari, 1998; Rutter, 1989). There has been a particular focus on the effects of psychological disorders amongst women in the postnatal period because infancy may be a time that the child is particularly vulnerable because of the rapid development which is taking place. Most of this work has concentrated on maternal depression (Cummings & Davies, 1994; Downey & Coyne, 1990; Murray & Cooper, 2003) and to some extent eating disorders (Agras, Hammer, & McNicholas, 1999; Patel, Wheatcroft, Park, & Stein, 2002). This research has generated considerable evidence that these disorders compromise maternal sensitivity during mother–infant interaction, and that the disturbance is associated with poorer child outcomes.

Given that anxiety disorders are common amongst women of child bearing age (Kessler, Keller, & Wittchen, 2001), it is surprising that little research has focused on the potential impact of maternal anxiety on parenting and child development. There is a body of research which has focused on anxious children and the quality of parenting they receive (see review by Wood, McLeod, Sigman, Hwang, & Chu, 2003) and there are a few reports of the relationship between mothers with anxiety disorders and their children, typically aged 6–15 years. These have shown that maternal anxiety disorder gives rise to changes in parenting in the form of reduced productive engagement and more withdrawn or disengaged behavior (Woodruff Borden, Morrow, Bourland, & Cambron, 2002), altered emotional climate (Turner, Beidel, Roberson-Nay, & Tervo, 2003), and reduced warmth and granting of autonomy and increased catastrophizing (Whaley, Pinto, & Sigman, 1999). In an expansion of the Whaley et al. study, these parenting variables were found to be associated with both the anxiety status of the child and that of the mother (Moore, Whaley, & Sigman, 2004).

As far as we are aware, there are no published reports examining the nature of the interactions between anxious mothers and their infants. Such studies are important for a number of reasons. First, as already highlighted, the infant is developing rapidly and may be particularly susceptible to parenting difficulties. Second, it is important to know if parenting difficulties demonstrated amongst anxious parents of older children are evident in the early years of life. Third, in attempting to disentangle bidirectional effects on interaction, the postnatal period is important, as any interactional difficulties are more likely to stem from within the parent and are less likely to be as a consequence of child anxiety, although infant temperament factors may influence parenting (Kagan, Snidman, McManis, & Woodward, 2001).

The aim of this study was to investigate the impact of maternal anxiety on the maternal contribution to mother–infant interaction, using video recorded observation of play in a sample of high trait anxious and non-anxious mothers and their infants. We hypothesized that anxious mothers would exhibit reduced sensitivity and facilitation during interaction. We chose these as our principal outcome measures as they have been shown to be key factors mediating the adverse effects of parental psychological disorder on child development (Murray & Cooper, 2003).

## 1 Method

### 1.1 Participant characteristics

High-trait anxiety (*n* = 32) and moderate to low-trait anxiety (*n* = 32) mothers with infants aged 10–14 months were recruited using advertisements in health centers and postnatal group newsletters. Of those women (*n* = 106) who expressed interest and received information, 64% (*n* = 68) returned consent forms and were screened with the trait version of Spielberger State-Trait Anxiety Inventory (STAI-T Spielberger, 1983). Participants scoring 40 or above were assigned to the high anxiety group (corresponding to the 70th centile, or above). Participants scoring 34 or less were assigned to the low anxiety group (corresponding to the 50th centile, or below).

All mothers were in a cohabiting relationship. As can be seen from Table 1, the two groups were comparable in terms of maternal and infant age, education, current work status, infant gender and parity.

### 1.2 Measures

#### 1.2.1 Instruments

The Spielberger State-Trait Anxiety Inventory (Spielberger, 1983) is a widely used 40-item schedule that indexes both trait (STAI-T) and state anxiety (STAI-S). It has strong psychometrics including established reliability (0.86–0.92) and validity.

The Edinburgh Postnatal Depression Scale (EPDS) is a 10-item questionnaire developed specifically to screen for depressive symptomatology in the postpartum period (Cox, Holden, & Sagovsky, 1987). The scale has been shown to be internally consistent with high levels of sensitivity and specificity (Murray & Carothers, 1990). Depression is known to be comorbid with trait anxiety in the postnatal period (Heron et al., 2004) and to influence maternal sensitivity (Murray & Cooper, 2003). Therefore the EPDS was included so that analyses could be conducted to minimize the possibility that any differences in mother–infant interaction between anxiety groups were due to differences in depressive symptomatology.

#### 1.2.2 Videotaped interactions

Each mother and infant pair was videotaped in their home. After an initial 2.5 min of free play without toys (mothers were invited to play a clapping game or to chat or sing with their infant, primarily to distract the infant's attention away from the camera and to allow for a period of acclimatization to being filmed), four toys of varying difficulty were presented sequentially for 2.5 min each, forming the 10-min rated as an outcome measure. Mothers were requested to play with the toys in any way they liked, the toys not being a test of ability. The same toys (stacker ring, hammerballs, musical toy and shape sorter) were chosen for age-appropriate infant exploratory play and have been used in previous research (Stein, Woolley, Cooper, & Fairburn, 1994). Considerable effort was made to help mothers feel at ease and to get used to the camera, in an attempt to reduce situational anxiety.

#### 1.2.3 Observer measures of interaction

All videotapes were rated by a coder who was blind to the psychological status of the mother. The coding schedule was based on schedules used in work by Stein et al. (1994), modified with a rating scale to measure maternal focus of attention.

The time-sampled variables, all rated on a 1–5 scale for each 2.5 min section of play were:

• *facilitation*: a measure of maternal behavior or verbal encouragement which assists the infant in an activity in which he or she is already engaged, or seems ready to engage, without taking over. Facilitation requires that the mother notices what her infant is doing, wanting or attempting to do, and gauges when and how assistance or encouragement is needed, e.g. positioning a toy to enable the child to reach or hold it;
• *sensitivity*: the degree to which a mother interprets the infant's clear and definite signals accurately and responds to them appropriately and promptly. This scale is an adapted version of Ainsworth's sensitivity scale (Ainsworth, 1969);
• *maternal emotional tone*: a primarily behavioral measure of positive maternal emotion based on posture, facial expression and tone of voice;
• *maternal focus of attention*: a measure of the total time mother's overall focus (gaze) was solely on the child or the activity the child was involved with;
• *infant emotional tone*: a behavioral measure of infant positive emotion.

The event-sampled variables for each 2.5 min section of play were:

• *intrusions*: actions that cut across, take over or disrupt the child's activity;
• *non-contingent utterances*: maternal utterances that are not in context, incongruous or non-contingent with the interaction or the child's behavior.

A random sample of 20% of tapes was independently rated by the first author to assess inter-rater reliability. Cohen's weighted Kappa values for time-sampled continuous variables ranged from 0.62 to 0.76, that is, good to very good (Altman, 1991), with the exception of maternal focus of attention (*κ* = 0.58). For event-sampled behaviors, the percentage concordances were 96% and 99%.

### 1.3 Procedure

The assessments took place in the mother's home. After written informed consent was obtained, demographic information was collected and the dyads were videotaped playing with the predetermined sequence of toys. Participants then completed the STAI-S and EPDS. Participants received a small gift token (£10, approximately $17) and a copy of the videotape.

## 2 Results

Variables were formally screened for normality using the Shapiro–Wilk test. Sensitivity, facilitation and state anxiety were normally distributed, with all other interaction outcome variables being positively skewed even after transformation. Therefore these variables were subject to non-parametric analysis.

Examination of correlations between variables indicated that sensitivity and facilitation were so highly correlated (*r* = .92) that these two variables were collapsed to create a composite variable named sensitive responsivity.

The characteristics of the sample are presented in Table 1. As evident, relative to the low anxiety group, trait anxiety, state anxiety and depression were higher in the high trait anxiety group.

Table 2 displays observer measures of mother–infant interaction. These are presented as mean ratings across all four tasks as a repeated measures ANOVA revealed no effect of toy order.

The high trait anxiety group of mothers exhibited lower levels of sensitive responsivity (*p* = .03, effect size = 0.59), and maternal emotional tone (*p* = .01, effect size = 0.54). Although non-contingent utterances occurred rarely, with seven participants accounting for only 11 occurrences in total, there was a significantly increased level of non-contingent utterances in the high anxious group (*p* = .045). No differences between groups were found on maternal focus of attention, infant emotional tone or intrusions.

In an attempt to minimize the possibility that the observed differences could be accounted for by the higher levels of depressive symptomatology in the high trait anxiety group, comparisons were made of subsamples from each group, differing in trait anxiety but not depression. In order to create a subsample low in depressive symptomatology within the high trait anxiety group, a median split on EPDS score was performed. Those scoring below the median (the median EPDS score for the high trait anxiety group was 8.5) formed the high trait anxiety/lower EPDS subsample (*n* = 16). To create a subsample comparable on depressive symptomatology within the low trait anxiety group, a median split on EPDS score was performed, with those scoring at or above the median (the median EPDS score for the low trait anxiety group was 3) forming the low trait anxiety/higher EPDS subsample (*n* = 20). These two subsamples remained significantly different on STAI-T (*p* < .001) and STAI-S (*p* = .001), but were comparable on EPDS (high anxiety mean = 5.75, low anxiety mean = 5.20, *t* = 0.69, NS) and all demographic variables. When the analyses of observer measures of interaction were rerun, the same pattern of differences was found. Thus the high trait anxiety/lower EPDS subsample exhibited lower levels of sensitive responsivity (*p* = .018), and maternal emotional tone (*p* = .049) and more frequent occurrence of non-contingent utterances (*p* = .04). As before, no differences between groups were found on maternal focus of attention, infant emotional tone or intrusions.

## 3 Discussion

The present study aimed to investigate the impact of maternal anxiety on the maternal contribution to mother–infant interaction, in a community sample, by comparing groups of high trait anxious and non-anxious mothers together with their 10- to 14-month old infants. We hypothesized that parenting tasks requiring sensitive responsivity would be compromised by high anxiety. Blind rated observer measures of interaction revealed reduced maternal sensitive responsivity (effect size 0.59), and lower emotional tone (effect size 0.54) in high trait anxious mothers. [There was no difference in infant emotional tone between groups, suggesting that the differences in maternal behavior were probably not accounted for by infant emotional state.] These findings are broadly consistent with and extend findings of altered patterns of interaction between anxious parents with their school-age children which demonstrated less effective engagement (Woodruff Borden et al., 2002) and reduced warmth (Whaley et al., 1999). As anxiety is often comorbid with depression in the postnatal period (Heron et al., 2004) we conducted a further subsample analysis, comparing two samples, one high and one low on trait anxiety but with both exhibiting relatively low levels of depressive symptomatology. The same pattern of interactional differences was found between these subsamples, providing support for the view that differences between the two anxiety groups were not accounted for by depressive symptomatology.

We also found an increase in the incidence of non-contingent comments in the group of high anxious mothers, despite there being no overall difference in maternal focus of attention. However, as these were infrequently occurring events, caution needs to be exercised in interpreting this result.

This is the first study to demonstrate differences in mother–infant interaction in high trait anxious compared to low trait anxious mothers of infants. We report findings from a relatively small community sample not at high risk for psycho-social adversity, thus minimizing the effect of potential confounding variables and allowing specific focus on the possible role of anxiety in relation to mother–infant interaction. Finding such differences in interaction evident in an analogue sample selected by trait anxiety, rather than diagnosis of anxiety disorder, suggests that the effects of maternal anxiety on mother–infant interaction in a clinical sample may be substantial.

This study was conducted within participants’ homes to increase the ecological validity of the findings, although it should be noted that this may have contributed to the difficulties in recording the direction of maternal attention for further fine-grain analysis. It will be important to replicate the findings in a larger sample of clinically anxious mothers and their infants, especially as larger samples would allow for analyses to partial out the differential effects of anxiety and depression. Further studies to determine the impact of maternal anxiety on mother–infant interaction are particularly warranted, as the earlier mother–child interaction is investigated, the less likely that child characteristics make a contribution to interactional disturbances, as the child will not have developed anxiety per se, although it may have increased fear responses or behavioral inhibition (Craske, 1999; Hudson & Rapee, 2004). The focus on the mother's contribution to interaction in this study is not intended to understate the infant's contribution to the reciprocal nature of parent–infant interactions, particularly in light of increasing evidence of bidirectional effects on interaction (Moore et al., 2004). Further studies would be strengthened by including measures of infant behavior, temperament and neurophysiology (Manassis & Bradley, 1994; Vasey & Dadds, 2001) to exclude the possibility that altered patterns of interaction are accounted for by infant factors.

The results reported need to be interpreted cautiously due to several limitations. The findings we report are from a relatively small sample of predominantly Caucasian and largely middle-class mother–infant dyads. Further studies are required to test the generalizability of the findings in samples with greater demographic diversity. As this study did not include a diagnostic interview measure of anxiety, it is not possible to determine the impact of anxiety of clinical severity on mother–infant interaction. However, as already noted, the present results suggest that the effects of maternal anxiety on mother–infant interaction in a clinical sample may be substantial.

One of the key questions is *why* anxious mothers demonstrated lowered levels of sensitivity. Given the design of the study it is only possible to speculate on underlying mechanisms. Two mechanisms in particular seem plausible: first, anxiety leads to an increased focus on negative threat related issues (Williams, Watts, MacLeod, & Mathews, 1997) and adversely affects attentional processing (Mogg, Mathews, Eysenck, & May, 1991). These disturbances may in turn interfere with maternal sensitive responsiveness. Second, anxiety disorders, when associated with insecure attachment representations may adversely affect attention and memory processes also potentially affecting mother–infant interaction (van Emmichhoven, van IJzendoorn, de Ruiter, & Brosschot, 2003).

In conclusion, this study provides evidence of the impact of maternal anxiety on mother–infant interaction in the postnatal year and adds to the body of studies reporting the impact of maternal anxiety on mother–child interaction in older children.

## Figures and Tables

**Table 1:** Demographic variables and characteristics of participants

	High anxious group (*n* = 32)	Low anxious group (*n* = 32)	*t* (d.f.)
Age (years)	33.20 (4.78)	33.62 (4.04)	0.39 (62)
Infant age (months)	12.03 (1.28)	11.79 (1.30)	−0.72 (62)
STAI trait	48.84 (7.91)	29.44 (2.91)	−13.03 (62)**
STAI state	35.68 (7.01)	25.22 (4.11)	−7.26 (61)**
EPDS	9.59 (5.27)	3.59 (2.95)	−5.62 (62)**

	High anxious group (*n* = 32)	Low anxious group (*n* = 32)	*χ*^2^ (d.f.)
Education (degree)	*n =* 22 (69%)	*n =* 23 (72%)	0.08 (1)
Current work	*n =* 19 (48%)	*n =* 21 (66%)	0.27 (1)
Infant gender	Male = 18 (56%)	Male = 11 (34%)	3.09 (1)
Parity	First born = 19 (59%)	First born = 17 (53%)	0.25 (1)

** *p* < .01.

**Table 2:** Observer measures of interaction

	Mean (IQR^a^)		Z^b^	*p*-Value
	High anxious group *(n* = 32)	Low anxious group *(n* = 32)		
Time sampled (1–5)				
Sensitive responsivity	3.43 (0.75)^c^	3.82 (0.56)^c^	2.36 *t* (62 d.f.)^d^	.021*
Maternal emotional tone	3.66 (3.50–4.0)	3.86 (3.75–4.0)	2.48	.01*
Maternal focus on infant	4.83 (4.75–5.0)	4.83 (4.75–5.0)	0.61	.54
Infant emotional tone	3.23 (3.0–3.44)	3.15 (3.0–3.25)	−1.13	.26

Event sampled				
Non-contingent utterances	0.78 (0–0)	0.01 (0–0)	2.01	.045*
Intrusions	0.07 (0–0)	0.03 (0–0)	−0.86	.39

a Interquartile range.

b Mann Whitney test.

c Mean (S.D.).

d Independent samples *t* test.

* *p* < .05.
